# Integral Healthcare: The Benefits and Challenges of Integrating Complementary and Alternative Medicine with a Conventional Healthcare Practice

**DOI:** 10.4137/imi.s2239

**Published:** 2009-10-19

**Authors:** Christina L. Ross

**Affiliations:** Candidate in Energy Medicine, Akamai University, Hilo, Hawaii. Email: cross@triad.rr.com

**Keywords:** complimentary medicine, alternative medicine, healthcare

## Abstract

Today’s medicine is in the midst of an undeniable crisis. Calls to reform healthcare are in the forefront of economic and political discussions worldwide. Economic pressures reduce the amount of time physicians can spend with patients contributing to burnout among medical staff and endangering the patient iatrogenically. Politicians are getting involved as the public is calling for more affordable healthcare. A new paradigm must be embraced in order to address all aspects of this dilemma. It is clear that science and technology have resulted in vastly improved understanding, diagnosis, and treatment of disease, but the emphasis on science and technology to the exclusion of other elements of healing has also served to limit the development of a model that humanizes healthcare. The healing of a patient must include more than the biology and chemistry of their physical body; by necessity, it must include the mental, emotional and spiritual aspects. Because of these challenges, the development of an integral healthcare system that is rooted in appropriate regulation and supported by rigorous scientific evidence is the direction that many models of integrative healthcare are moving towards in the 21st century.

## What is Integral Medicine?

Integral medicine recognizes that human beings possess emotional, mental and spiritual dimensions that are essential in the diagnosis and treatment of disease and the cultivation of wellness. Integral medicine is about being concerned with the whole person rather than the disease; calling on the universal life force (prana or chi) manifested mentally, physically and spiritually. “Body, mind and spirit are operating in self; culture and nature, and thus health and healing sickness and wholeness are all bound up in a multidimensional tapestry that cannot be cut into without *loss*” (Schlitz,^1^ forward, p. *xl*).

According to philosopher Ken Wilber, there are four elements necessary for the progress of an Integral medicine paradigm: 1) *An expanded consciousness* which includes inner thoughts, feelings and spiritualism as well as outer behavioral indices such as race, culture, religion, sexual orientation and family influences. These stages of consciousness span a spectrum from sensory to mental to spiritual. Potentials coming from the physical, emotional, mental and spiritual spectrums effectively resist reductionism. Since we all have physical, emotional, mental and spiritual dimensions of being and awareness, attempting to reduce mind and spirit to matter can be considered folly; 2) *Holism*, which includes oneness of experience. Instead of an egocentric viewpoint or even an ethnocentric viewpoint, this paradigm embraces a worldcentric point of view; 3) *Intentionality*, which would integrate mind/body systems, and 4) *A larger self view* of the interconnectedness of all life. This view would create a larger consciousness, which in turn would manifest a larger self and then generate larger medicine. “An integral medical practice is a practice that makes room for the entire panoply of effective treatments across all quadrants and dimensions of human health and illness” (Wilber,^2^ forward p. *xxx*).

## How do we Progress from a Patho-Physiological System to an Integral Healthcare System?

Integrating healthcare is somewhat like getting drivers to switch from combustion engine automobiles to electrically powered motor cars. It is very difficult to “turn off the gas” and have the driver feel safe and confident that he will reach his destination. The most proficient way to move forward is with small steps. The transition from gas-powered automobiles to gas-free automobiles must go through a hybrid stage in order for drivers to feel comfortable with the new system. The same can be said about transitioning from a pathophysiological based medical system to an integral based system. There are many biases towards complementary and alternative practices that prevent total acceptance in the current medical field. Biases towards viewing spirituality as organized religion and also biases as to whether or not humans really have a conscious mind that is capable of healing the body on its own.

A lack of “qualified” research is also of major concern to many conventional medical practitioners. A biological based medical practice steeped in treatments proven through the “double-blind randomized controlled trial” (the gold standard of medical research) will need to be reassessed as a research method for complementary and alternative treatments (which have been time tested and take into account the whole person) comes to fruition.

## What Model can be Used for Integrating a Conventional Medical Practice?

A model for integrating medicine and psychology is already in existence can be expanded upon for an integral healthcare model. The Model for Integrating Medicine and Psychology (MI-MAP) was developed over a ten year period of training physicians in both behavioral health and psychological factors, and training psychologists in physical health and pathophysiology.^3^

MI-MAP attempts to combine the categorical and individual aspects of other models and create a more integrated depiction of factors to be assessed. It also organizes a sequential process by which the clinician can perform a comprehensive yet expedient inquiry regarding symptomatology relevant to the biopsychosocial model. This model serves as a guide to employ the concepts of the biopsychosocial model, and apply these concepts consistently with the process of clinical evaluation, treatment planning and clinical intervention. The agenda for developing the MI-MAP stemmed from several observations in the clinical training setting. This model could be extended to include spiritual imbalances as well as mental and emotional disorders.

Physicians and nurses often struggle with the psychosocial components of a biopsychosocial practice. In the medical setting, patients present their physical symptoms and physicians often focus exclusively on the diagnostics of the physical disease. The MI-MAP grew from the need to make attention to psychosocial factors easier yet more comprehensive for physicians, nurses, health psychologists, general psychologists and social workers, starting from the patients’ presenting complaints of physical symptoms.^3^ Incorporating spiritual counselors and alternative medicine practitioners will help with assessing all dimensions of the patient’s healthcare needs.

A check list of red flags pointing to biological, spiritual and mental health issues can be quickly diagnosed and attended to by all types of health practitioners. Here are ways to empower practitioners as well as patients to remain alert for signs and symptoms in all types of healthcare. This can also be advanced through a network of support groups for practicing primary care physicians, behavioral health practitioners, complementary and alternative practitioners and spiritual counselors.

## How do we Begin the Movement Towards Change?

Starting with educating physicians, the need to integrate complementary and alternative practices in medical schools is of utmost importance. According to Ken Wilber,^2^ over two thirds of medical schools now have courses in complementary and alternative medicine; however, the challenge is to educate by cross training spiritual and behavioral healthcare practitioners as well as medical practitioners. An integrally-informed medical practice educates the practitioner’s first so they can then decide which of the treatments—conventional, complementary, alternative, and/or holistic they wish to use. It is important to note that some integrative practices will *complement* Western medicine and some will provide an *alternative* to Western medicine. An integrative system of complementary and alternative medicine differs from a system which is considered integral. Integral medicine blends Western allopathic medicine with complementary and alternative medical choices. From these options the practitioner could then individually or collectively practice medicine instead of either ignoring or guessing at the causes of psychological, emotional or spiritual imbalances. “The challenge with many alternative, complementary or holistic practices is that for all their sincere efforts, their practices often create a *grab bag* of treatments from which a conventional medical doctor would have no idea which would work with her patient” (Wilber,^2^ forward p. *xxx*).

The transpersonal practitioner, however, knows when to refer out a patient, making room for new techniques by becoming part of a medical group or center that specializes in integral treatments. The transpersonal practitioner refers to a human-to-human connection that goes beyond the personal, physical ego self and connects with a deeper, more spiritual, transcendent, consciousness based practice.

Without primary care physicians having to become psychologists or spiritual counselors, there are ways to alert all types of health practitioners to spot red flags in each others’ scope of practice. Physicians may not be adept at all healing aspects of the human being, but they should at least be cognizant of psychological and spiritual issues in their patients and should be aware of the implications of ignoring these aspects when it comes to health and healing.

## Why is a Spiritual Aspect of Healing Necessary?

Today, spirituality is considered an important part of secular life. According to Norman Shealy, M.D., PhD.,^4^ “Concomitant with the spiritual decay of the Vietnam War, the redeeming influence of the humanistic psychology movement was born. It was followed by the transpersonal psychology movement, and then the holistic health and medicine movement.” Humanistic psychology emphasizes the importance of the individual, of feeling, of self-actualization. The transpersonal psychology movement emphasizes a connectedness with spirit, soul and God. The holistic health movement emphasizes the importance of the spiritual aspects of life in overall well-being.^4^

A health challenge or healing crisis opens for many people the realm of the spiritual. When the gift of good health is interrupted, we have the opportunity to realize that the well-being we tend to take for granted is a gift. The loss of good health is very often experienced as a gift because it can be a doorway to a new understanding of self in relationship to others. A whole new sense of gratitude for life can change a patient’s quality of life.

Spiritual counseling often involves a search for meaning in terminal illnesses, confronting suffering and exploring forgiveness and gratitude. Mind-body medicine focuses on the potential for mental, emotional, social, behavioral and spiritual processes that affect health and personal growth. Yoga, meditation, prayer and expressive arts such as dance, art and music are often suggested in mind-body practice.

## How can Conventional and Integrative Medicine Benefit from an Integral Model?

Of great concern are the growing number of uninsured persons and the lack of reasonable mental, emotional and spiritual healthcare coverage worldwide.^5^ The way insurance is relegated is perhaps one of the biggest underlying deterrents in using the integral healthcare model. Conceivably the answer lies in moving from a carve-out system to a carve-in structure of managed behavioral healthcare. Insurance issues in the healthcare industry remain as big a problem as the reductionistic theory of matter in medicine. In most cases insurance benefits for a specific service category are separated from other insurance benefits and managed under a different contract. So even if medical doctors decided to practice alongside psychologists, psychiatrists and spiritual counselors, the insurance processing systems would be separate. This carve-out structure system increases the chances of over prescribing psychotropic medications by primary care physicians who may not be trained in such unless they are a psychiatrist.^3^

There is also a lack of equality granted to mental health coverage as compared to conventional medical coverage. According to Marilyn Schlitz,^1^ 60 to 70 percent of all medical visits primarily have a psychosocial basis and 25 percent of primary care patients have a diagnosable psychological disorder with anxiety and depression being the most common. Schlitz asserts that 50 percent of patients with mental health problems are seen only in primary care, and 70 percent of all psychotropic medications are prescribed by non-psychiatric physicians. Less than one third of adults with a diagnosable mental disorder receive treatment each year, leaving the majority without care.

Integrating medicine with a carve-out structure of managed holistic healthcare would greatly benefit both the patient and the physician, especially if equal coverage is granted for all modalities. “Integral medicines goes one step further: it treats the illness, the patient and the physician” (Wilber,^2^ forward, p. *xix*).

## So What is the Next Step Towards Initiating an Integral Medicine Practice?

If Western medicine could embrace a system where physicians, social workers, spiritual counselors and behavioral healthcare providers are able to collaborate more freely by co-locating, they would readily see how the quality of the care provided to their patients improves. This structure may have the added benefit of destigmatizing mental healthcare as patients come to see mental health providers as part of the medical team.

It would be quite simple to follow up a physical health exam with a mental and spiritual health visit if medical doctors, psychologists and spiritual counselors worked in the same location. Support groups could meet on site at the medical center. The patient could book an appointment with their healthcare practitioners in the same office and feel confident that all their needs are being met by the same medical team. Having to leave the office and find a psychologist or spiritual advisor who doesn’t know the patient’s medical history can give pause to the patient not following through with the recommended appointments. This model for integrating conventional medicine with psychology and spirituality would provide an expedient assessment sequence to assist psychologists and social workers in understanding the stresses and coping demands of various physical illnesses as well as to orient physicians and nurses to the psychosocial factors that may interfere with important medical outcomes.

Barriers to an integral system of this type include the fact that managed care has not provided financial incentives for adopting an integrated model of healthcare.^6^ There is a lack of integrated technologies making administrative integration difficult and most medical facilities are not structurally designed to accommodate behavioral health providers and are therefore prohibitive of co-location. The lack of adequate reimbursement in insurance policies is another problem and cost codes for mental health remain separate from the physical health system creating billing problems for the patient, doctor and insurance company.

If a more integrated approach were taken in health insurance and a managed care organization owned and operated its behavioral health organization as a subsidiary and would retain the risk for the mental health services, management could focus on the person as a whole as well as provide a combination of medical and psychological services. This is important since the World Health Organization^7^ ranks psychological disorders second only to cardiovascular disease as a leading cause of worldwide disability.

## Why Does Conventional Medicine Need Complementary and Alternative Therapies?

It is important to understand that integrative medicine cannot exist without both conventional and alternative therapies to draw from, which, in turn, supply the context for each other’s very existence. “We are all part of a health care hologram—shine a light through one of us, and you will see an image of the whole”.^8^ Larry Dossey, MD suggests that the popularity of alternative therapies and therapists is largely due to the fact that they help people find meaning in their lives when they need it most. Dossey writes, “No matter how technologically effective modern medicine may be, if it does not honor the place of meaning in illness, it may lose the allegiance of those it serves” (1982, page 154). Integrative medicine honors the body’s own intelligence and transformative nature.

“The net result of the problem between conventional and alternative approaches is that physicians and nurses are very unhappy with the present state of conventional medicine and yet they often distrust the holistic alternatives” (Wilber,^2^ forward, p. *xxxii*). They know conventional medicine is limiting them personally and in the healing they offer the sick, yet they may suspect that too many of the alternative and holistic approaches have abandoned sound research, scientific evidence and rigor in what amounts to a medical version of “popularity with the public”.

Misunderstandings with regard to integral medicine include the idea that it doesn’t just diagnose or fix—it uses the body’s innate ability to heal itself. Conventional medicine does not use the principle that consciousness shapes our understanding of health and disease. Because of this misunderstanding, integral medicine will require a reassessment of assumptions. Objectively it cannot exist—separate from our consciousness. Today’s medicine must integrate and appreciate multiple social and cultural approaches to healing. Its desire for health and healing is as important as scientific information and technology.

## What are the Cultural Benefits of an Integral System Versus a Conventional or Integrative System?

Different cultures add other dimensions to the art and science of healing. According to David Bohm,^9^ complex linkages of the “implicate order” between mental, physical, social, cosmic and spiritual realms provide the holistic phenomena that Navajo people refer to as indigenous medicine. Bohm’s notion of holistic order, in which any given element “contains the totality of the cosmos enfolded within itself ”, pertains to both consciousness and the Navajo healing process; hence it is relevant to noetics, the scientific study of phenomena classified under such descriptors as “consciousness” and “mind”.

“Westerners often emphasize the transcendental side of shamanism to the neglect of its practical aspects. In the Navajo way of thinking, the causes of imbalance, (disease) and healing (restoration of balance) areintrinsically interrelated. Other physical symptoms may attach themselves to the original imbalance. One becomes aware of a multiplicity of invisible interconnections within the organic system. These complex alignments are visible and understandable to Navajo diagnosticians. However, they are most often invisible and discounted by Western allopathic physicians who have been trained to deal with causality-factored diagnoses”

(Maryboy,^10^ p. 408).

Following this same holistic paradigm, Traditional Chinese Medicine uses Chi, the “vital energy of life”. Ancient people concluded that all things in the universe (with or without life) are made up by an ultimate, invisible, yet ever-existing Chi. Ancient medical theory posits that the human body is formed by Chi and that the body correlates with nature. The first complete Traditional Chinese Medicine textbook, *The Yellow Emperor’s Classic of Internal Medicine* originally published in China more than 2,000 years ago explains that diseases are believed to be caused by deficits and imbalances of Chi in the body. Different Traditional Chinese Medical treatments have the common objective of regulating Chi and restoring balance.

The ancient science of Tibetan medicine is rooted in the teachings of Buddha and the essence of these teachings is the central importance of the mind. The Buddha says the mind is both the source of happiness and the root of suffering. At the same time it possesses an extraordinary capacity for healing; it also plays a part in making us ill. “Tibetan medicine is an integrated system of healthcare that has served the Tibetan people well for many centuries; but the end result is that it doesn’t matter whether a patient utilizes Western medical services or the ancient healing methods; the most important thing is that the patient is an active participant in their own healing process” (Rinpoche,^11^ page 420).

## What Organizations are Moving Towards an Integral Model?

For an overall view of the integrative healthcare process, reference the American Holistic Medical Association (AHMA) which serves as the leading advocate for the use of holistic and integrative medicine by all licensed healthcare providers. The AHMA embraces integrative, complementary and alternative medicine techniques, while holding onto what is helpful in allopathic medicine—understanding that healing includes the body, the mind, the emotions, and the spirit.

“The AHMA is committed to increasing awareness and understanding of the natural healing tenets of Holistic Medicine and to link together those who wish to utilize and promote a holistic approach to conventional and integrative medicine. Teaching the value of prevention and wellness instead of a “quick fix” is best accomplished through correcting core imbalances and by addressing contributing factors”.

Other organizations providing information for integral healthcare include the National Center for Complementary and Alternative Medicine (NCCAM) and also the National Library of Medicine (NLM), which have partnered to create *CAM on PubMed*, a subset of NLM’s PubMed. PubMed provides access to citations from the MEDLINE database and additional life science journals. It also includes links to many full-text articles at journal Web sites and other related Web resources. Organizations that specialize in integrative resources include the National Cancer Institute (NCI), the National Institute of Arthritis and Musculoskeletal and Skin Diseases (NIAMS), the National Institute of Environmental Health Sciences (NIEHS), the Office of Dietary Supplements (ODS) and the Agency for Healthcare Research and Quality (AHRQ).

One example of an Integral healthcare model is the Health and Healing Center of the Institute of Health and Healing at the California Pacific Medical Center. The center integrates self-care with expert care and the best of conventional medicine with proven healing practices from around the world. The center’s physicians and practitioners are experts in the world’s great healing traditions, including Western medicine, all backed by research. The center offers a personalized approach to healthcare that combines conventional and complementary approaches. The Institute of Health and Healing also offers classes to teach individuals and practitioners fundamental tools for wellness.

Other treatment centers around the world have taken up the challenge of integral healthcare. One in particular is The Cancer Centers of America (see www.cancercenter.com), which uses mind/body medicine, guided imagery, holistic patient centered approaches, naturopathic medicine and spiritual support along side chemotherapy, surgery and radiation treatments. This integrated approach has shown to be rewarding to not only the patient, but also the healthcare practitioners. The testimonials regarding improvements in health rates and hospital admission/release/re-admission rates and lowering of mortality rates is substantial as well as showing an improved quality of life for the patient.

The Maricopa Integrated Health System (http://www.mihs.org), a burn center, is another integrated clinic and the Roger C. Lipitz Center for Integrated Health Care, a foundation of Johns Hopkins Bloomberg School of Public Health (www.jhsph.edu/LipitzCenter) has integrated conventional and alternative care as well.

Ways an integral health care model would help patient/doctor interaction include instructions on patient care and self-management as well as somatization (which includes underlying emotional causes for medical diagnosis such as chest pain, fatigue, dizziness, headaches and constipation). On average 85 percent of these illnesses were the result of psycho-social issues and undiagnosed and misdiagnosed mental illness.^3^

For the last several decades, behavioral scientists have demonstrated improved clinical outcomes as well as cost savings resulting from targeted psychological interventions that have been integrated within primary and specialty medical care. Many chronic diseases, such as diabetes, pulmonary diseases, hepatitis and asthma can be prevented or managed more effectively with behavioral interventions targeted at diet, exercise, smoking, chronic pain, sexual practices and adherence to medical regimens to improve medical treatment outcomes. Surveyed medical doctors also expressed an increased sense of confidence in their ability to provide patients the medical care they need when working together with behavioral care practitioners.^1^

Katon^12^ and colleagues demonstrated superior clinical outcomes for the treatment of depression in an integrated primary care setting with co-located mental health treatments provided along with primary medical care. After being referred to a mental health provider, 44 percent of patients showed symptom reduction. This study further revealed that 80 percent of participating physicians expressed that the collaboration with mental health providers greatly increased their satisfaction in healing depression.

Whereas much of the writing on the integral health care model focuses on aspects that are not emphasized in our contemporary culture, it does incorporate an evidence-based approach. Data from many scientific studies support the overall value and efficacy of various elements of integral health care. Research Professor Candace Pert, PhD^13^ explains how her work has shown that the form of neuropeptides and their corresponding cellular receptors, which make up our biological systems, are literally flooded by our cognitions and emotions. Neuropeptide receptors are not limited to the brain; they are present on cells in tissues throughout the body. Emotions are therefore a bridge between mind and body.

James W. Pennebaker^14^ and his students are exploring the links between traumatic experiences and physical and mental health. His most recent research focuses on the nature of language, personality and emotion in the real world. And Stephanie Simonton-Atchley^15^ is researching the growing evidence that psychosocial factors contribute to disease progression.

At times, the sociopolitical and economic dimensions of healthcare can seem overwhelming. There are so many interests at stake, and so many complex relationships between institutions and individuals. In the integral model, there is a shift from a disease centered approach to one that seeks to build optimal healing environments. What is being called for are organizations that align hospitals, physicians, nurses, alternative practitioners, and communities to optimize patient care. But the focus of a fully integral system also includes the workplace, the home, and the individual. It spans the distance from religion to culture, social interaction to family interaction. The public is calling for something more, and many of the leaders in hospitals and government recognize the need for whole system change.^1^

## Conclusion

Unless and until medicine embraces the paradigm of the patient as a mental, physical and spiritual being, the medical industry is destined to be stuck in an infinite quandary. The paradigm shift from integrative to integral medicine requires more than an evolution from the basic model of “body, mind and spirit” into a more inclusive social, political, economic, metaphysical, ecological and worldwide dimension of health care. “The multiple dimensions of the patient with self and others require equal status with the physical and objective foundations of science”.^1^ Although physicians may not be adept at healing all facets of the human being, they should at least be cognizant of cultural, social, spiritual and psychological issues in their patients and should be aware of the implications of ignoring these aspects when it comes to health and healing. Integral medicine is the next step up from integrative medicine because it incorporates not just conventional and alternative medicine, but all dimensions of healing—from physical to psychological and cultural to spiritual.
